# Dynamic trends in skin barrier function from birth to age 6 months and infantile atopic dermatitis: A Chinese prospective cohort study

**DOI:** 10.1002/clt2.12043

**Published:** 2021-07-03

**Authors:** Ying Ye, Piaoping Zhao, Limin Dou, Yi Zhang, Kaku Ken, Hongjian Gu, Yalan Dou, Wei Gao, Lingfeng He, Xiaotian Chen, Xiangyuan Huang, Lei Zhang, Yun Li, Liuhui Wang, Weili Yan

**Affiliations:** ^1^ Department of Clinical Epidemiology Children’s Hospital of Fudan University & National Children Medical Center Shanghai China; ^2^ Department of Dermatology Children’s Hospital of Fudan University & National Children Medical Center Shanghai China; ^3^ Department of Research & Development Pigeon Maternal and Infant Skin Care Research Institute Shanghai China; ^4^ Shanghai Minhang Maternal and Children Health Care Hospital Shanghai China

**Keywords:** atopic dermatitis, birth cohort, prospective, skin barrier, TEWL

## Abstract

**Background:**

Skin barrier functions develop after birth and may be related to skin disorders in infants. We aimed to assess associations between dynamic trends of four skin barrier functional parameters in early life with infant atopic dermatitis (AD).

**Methods:**

Based on the prospective cohort MKNFOAD (NCT02889081), we examined transepidermal water loss (TEWL), stratum corneum hydration (SCH), skin pH, and sebum content at five anatomical sites (cheek, forehead, forearm, abdomen, and lower leg) in 418 term infants at birth, 42 days, and 6 months. Trend differences by sex and association with AD at age 1 year were tested using variance analyses. Associations of the parameters with AD risk were tested using discrete time survival analysis, adjusting extensive covariates including parental history of allergy, infant’s sex, birth weight (kg), and delivery mode. Odds ratios (ORs) and 95% confidence interval (CIs) were reported.

**Results:**

Overall TEWL and SCH appeared trends of increase while skin surface pH and sebum content showed trends of decrease within the first six postnatal months. Sex differences were significant for sebum content only (*p* < 0.001). After adjustment for parental and children covariates, cheek TEWL at birth (OR = 1.26, 95% CI 1.00–1.57, *p* = 0.045) and 42 days (OR = 1.52, 95% CI 1.17–1.97, *p* = 0.002) were significantly associated with increased AD risk. Associations were not observed between SCH, skin pH, and sebum content at birth or 42 days with AD.

**Conclusions:**

Skin barrier functions of Chinese term infants varied nonlinearly after birth. Higher postnatal TEWL levels in early life indicate higher risk of early‐onset AD.

## 1 INTRODUCTION

The skin barrier has a protective role against the external world and in maintaining homeostasis. After leaving the womb, the skin of newborns undergoes active and physiological adaptation to the new environment and a transition to maturation within the first months of life.^1^, ^2^, ^3^ Infant skin functions are vulnerable to extrauterine triggers such as bathing and moisture, which implies skin incompetence^4^, ^5^ that is different from adults.^6^, ^7^, ^8^ Barrier dysfunction is related to certain skin conditions like atopic dermatitis (AD) or diaper rash in infancy.^9^, ^10^ Clarifying the adaptation of infant skin is of clinical importance for infant skin care and assessing the risk of developing skin disorders.

Previous studies have shown age effect in the infant skin transition course and the physiologic structure of the epidermis based on cross‐sectional study,^6^, ^11^, ^12^, ^13^ and skin properties vary with ethnicity and skin phototype.^14^, ^15^, ^16^ Cohort studies on the features of postnatal skin physiologic development were most from Caucasians,^5^, ^17^, ^18^, ^19^ studies in Asian were relatively limited in 19 and 24 infants respectively.^20^, ^21^ These studies were limited in either the lacking of skin disorder information or in small sample size, their results failed in showing the skin adaptation or maturation after birth in a general infant population. As skin barrier functions differ among anatomical sites,^22^, ^23^ measurements including exposed and unexposed sites will help to reveal regional variation in postnatal trends of skin physiologic development. To our knowledge, the dynamic change of location‐specific skin function has not been reported before especially on exposed and unexposed part.

Monitoring transepidermal water loss (TEWL), stratum corneum hydration (SCH), skin pH, and sebum content is used to evaluate skin function^24^, ^25^, ^26^ and the effects of intervention.^27^ There is evidence of a reverse association with AD of TEWL^28^, ^29^; however, the contribution of the other parameters is unclear. Quantitative description of these parameters during infancy will facilitate comprehensive clinical evaluation of infant skin status and identifying high‐risk infants among the general population.

Based on a birth cohort study in China, skin parameters (TEWL, SCH, skin pH, sebum content) were measured postnatally from birth to 6 months, and affected status of AD was assessed and diagnosed at the visit of at 1 year old. We described the dynamic change in skin parameters over the first 6 months of life in infants without AD. We assessed the association between skin barrier functions at early stage of life and the risk of early‐onset AD in the entire study population.

## 2 METHODS

### 2.1 Study design and participants

The study population was from the MKNFOAD birth cohort in Shanghai. The MKNFOAD protocol was approved by the institutional ethnic review board (approval number: 2016‐34) and was registered at www.clinicaltrials.org (NCT 02889081). Signed informed consent was obtained from the legal guardians of participants before data collection began.

Infants in the cohort meeting the following criteria were included in this study: (1) born full term (gestational age ≥37 weeks); (2) underwent at least one assessment of skin barrier function at birth, at day 42, and 6 months postnatally; (3) followed up to age 1 year. Infants diagnosed with a congenital disorder of the skin or appendages were excluded. Infants received skin barrier function tests at birth, 42 days, and 6 months by trained nurses. Parents consulted with a dermatologist once their baby developed any skin symptoms, up to age 1 year.

Infant demographic information was extracted from the electronic medical records, including sex, birth weight, gestational age, delivery mode, and birth season. Body weight and height were measured at each follow‐up visit. Maternal and paternal histories of allergy, breastfeeding, and solid food introduction within 6 months were collected in interviews with infants' parents.

### 2.2 Assessment of skin barrier function

Four skin barrier function parameters were evaluated (TEWL, SCH, skin surface pH, and sebum content) at five anatomical sites (forehead, cheek, volar forearm, abdomen, and dorsal lower leg). An MPA4 multi probe adapter systems (Courage & Khazaka electronic GmbH) was used with probes (Tewameter TM300, Corneometer CM825, Skin‐pH‐Meter PH905, Sebunmeter SM815; Courage & Khazaka) directly contacting the skin surface. TEWL was examined with a probe contacting the skin surface continuously for 20 s. TEWL values for analysis were calculated based on the mean of readings from the most stable 5 s. Three readings of SCH were obtained and the mean was calculated for each body site. Skin pH was tested by direct touch at the test site. Sebum content was tested for 20 s continuously, only on the forehead.

Skin barrier function testing was conducted in maternity wards (within 12–96 h after birth) and at clinics (age 42 days and 6 months). Before evaluation, infants were acclimated to the controlled environment for at least 15 min. We excluded infants who were crying or sweating. Skin tests were conducted under stable temperature (23–26°C) and relatively humidity (35%–60%). Skin tests at birth were performed before routine bathing with no visible vernix caseosa on the test site. Regular calibration of each probe was conducted approximately every 15 days, according to instrument instructions.

### 2.3 Assessment of infant AD

Diagnosis of AD was based on Williams' criteria.^30^ Infants with suspected AD who had atypical symptom onset were treated by a pediatric dermatologist and followed up for subsequent assessment of symptoms. Final diagnosis was confirmed at age 1 year by three experienced pediatric dermatologists through panel discussion.

### 2.4 Statistical analysis

Statistical analyses were performed using Stata 15.0 (StataCorp LLC, College Station). Continuous data are presented as mean with standard deviation (SD) or median with interquartile range (IQR), as appropriate; categorical data are presented as absolute number with percentage. Comparisons of the general characteristics of infants with and without AD were performed using chi‐square or *t*‐tests. We described the distribution of skin barrier function parameters according to body site and age at follow‐up visits among infants who did not develop AD.

Mixed models for repeated measures were used to test the overall varying trends of skin barrier parameters over time and sex differences in the non‐AD group only at selected body sites (cheek and forearm only). We further performed these analyses in all participants to investigate overall trend differences between the AD and non‐AD groups by including an interaction term of the time and group (defining AD or not). The overall differences in the parameters over time between AD and non‐AD patients were analyzed by “contrast” and the estimations and 95% CI for each time points by “margins”.

To investigate the associations of these parameters with infant AD risk, we selected the cheek to represent exposed body sites and forearm to represent less‐exposed body sites. Sebum content was tested only on the forehead. Selected skin barrier parameters were normalized to Z values according to age‐specific means and SDs of the entire cohort. Discrete time survival analysis was performed to assess the associations of four skin parameters at birth and age 42 days with the risk of infant AD incidence by 1 year old. Time to incident AD was precised to month. First, we performed univariate analyses and significant associations were further analyzed using multivariate analysis, by adjusting for possible covariates including parental history of allergy, infant’s sex, birth weight (kg), birth season, delivery mode, postnatal breastfeeding, and solid food introduction within 6 months. The associations are reported as odds ratio (OR) and 95% confidence interval (CI); *p* < 0.05 was considered statistically significant.

## 3 RESULTS

### 3.1 Participant characteristics

**FIGURE 1:**
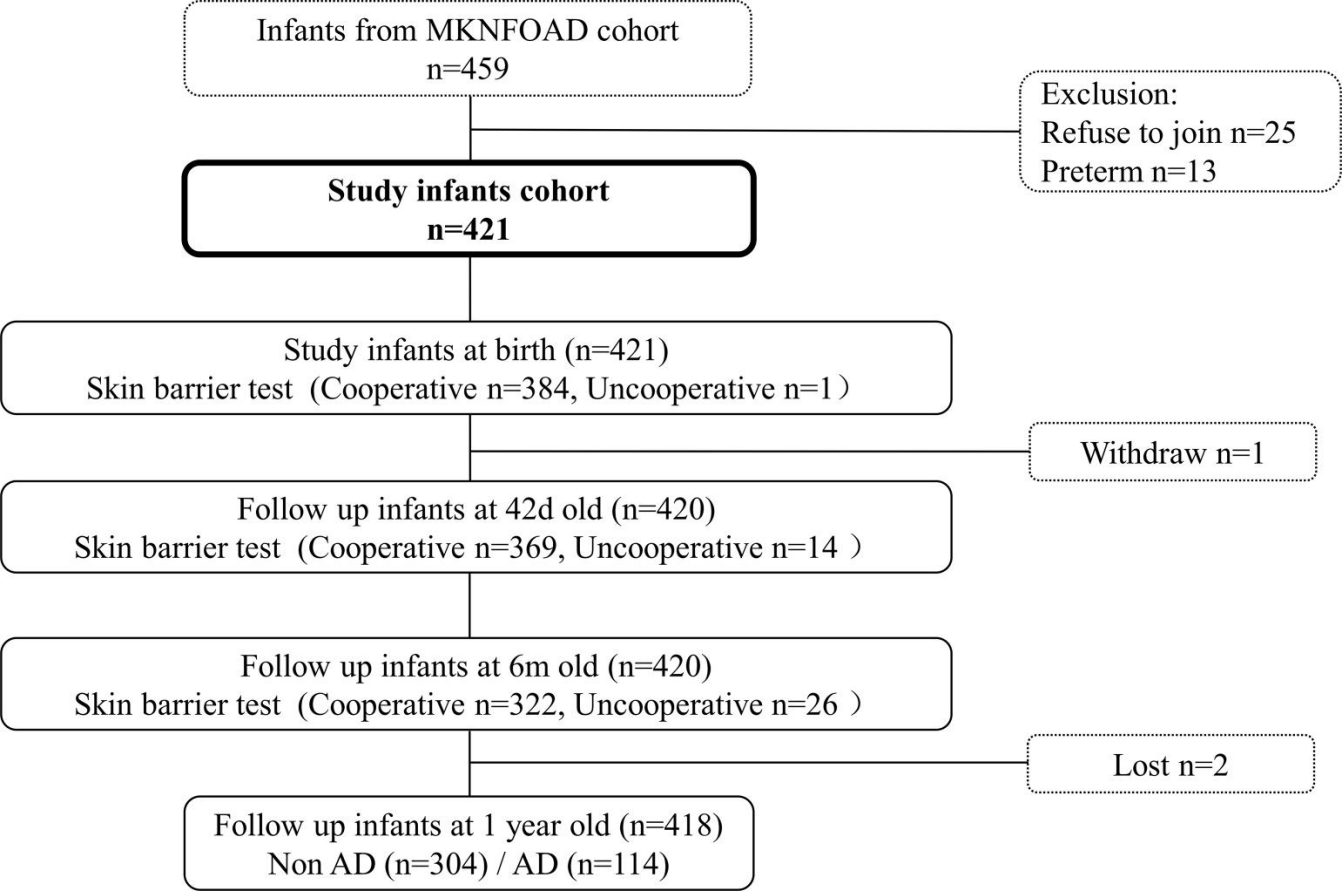
Flowchart of participants in the cohort. AD, atopic dermatitis

As shown in Figure 1, 418 infants were included in this study, with 219 (52.4%) boys. In total, 114 (27.3%) infants were diagnosed with AD within the first 1 year; the other 304 infants comprised the non‐AD group.

Infants were born at 39.3 ± 1.0 weeks with half by caesarean delivery; 83.3% were breastfed, and 54.6% started solid food before age 6 months. Compared with the non‐AD group, the AD group showed a generally larger body weight and height across the first 6 months; maternal and paternal allergy history was also more often reported among infants with AD (30.7% vs. 17.4%; 22.8% vs. 15.1%) (Table 1).

**TABLE 1:** General characteristics of infants by AD status

Characteristics	Overall	AD Group	Non‐AD group	*p* value
N	418	114	304	
Male	219 (52.4)	73 (64.0)	146 (48.0)	**0.004**
Gestational ages (week)	39.3 ± 1.0	39.3 ± 1.0	39.2 ± 1.0	0.52
Caesarean	212 (50.7%)	66 (57.9%)	146 (48.0%)	0.07
Birth weight (g)	3403 ± 371	3496 ± 395	3368 ± 356	**0.002**
Weight aged 42 days (g)	5076 ± 544	5210 ± 589	5026 ± 519	**0.003**
Height aged 42 days (cm)	56.5 ± 2.1	57.1 ± 1.9	56.3 ± 2.1	**<0.001**
Weight aged 6 months (g)	8465 ± 1088	8627 ± 1073	8403 ± 1090	0.06
Height aged 6 months (cm)	68.3 ± 2.2	68.8 ± 2.0	68.2 ± 2.2	**0.011**
Breast feeding	343/412 (83.3%)	89/113 (78.8%)	254/299 (85.0%)	0.14
Solid food induction within 6 months	225/412 (54.6%)	65/113 (57.5%)	160/299 (53.5%)	0.51
Family allergy history				
Mother	88 (21.1%)	35 (30.7%)	53 (17.4%)	**0.003**
Father	72 (17.2%)	26 (22.8%)	46 (15.1%)	0.06

*Notes:* Data shown are mean ± standard deviation or number (%), as appropriate. Chi‐square or *t*‐tests were used for the comparison between AD and non‐AD groups.

Abbreviation*:* AD, atopic dermatitis.

### 3.2 Dynamic trends of skin barrier functional parameters in infants from birth to age 6 months based on non‐AD group

The values of TEWL, SCH, skin pH, and sebum content varied across the time points at birth (36 ± 22 h), 42 days (44 ± 5 days), and 6 months (6.1 ± 0.2 months) for all five body sites (Table 2). From birth to age 6 months, dynamic changes appeared an overall increase trends for TEWL and SCH but a decline trends for skin pH and sebum content with age.

**TABLE 2:** Skin barrier parameters in the non‐AD group for different anatomical sites at birth, 42 days, and 6 months

Anatomical part	Visit	TEWL (g/m^2^/h)	SCH (a.u.)	Skin pH	Sebum content (ug/cm)
Cheek					
	Birth	9.35 ± 3.98	26.3 ± 10.3	6.07 ± 0.61	‐
	42 d	17.01 ± 7.83	36.9 ± 13.2	5.67 ± 0.43	‐
	6 m	8.00 ± 2.66	53.6 ± 12.7	4.98 ± 0.45	‐
Forehead					
	Birth	8.59 ± 5.16	23.4 ± 9.5	5.74 ± 0.83	69 (37,109)
	42 d	12.12 ± 6.00	40.6 ± 14.3	5.08 ± 0.56	58 (36,87)
	6 m	8.91 ± 4.00	60.5 ± 11.4	4.38 ± 0.30	5 (1,14)
Volar forearm					
	Birth	7.43 ± 3.49	17.0 ± 5.4	6.49 ± 0.68	‐
	42 d	10.18 ± 4.95	41.4 ± 9.9	5.03 ± 0.36	‐
	6 m	9.92 ± 3.62	53.8 ± 10.7	4.58 ± 0.21	‐
Abdomen					
	Birth	6.04 ± 2.40	22.4 ± 7.3	6.59 ± 0.68	‐
	42 d	8.57 ± 3.26	36.2 ± 8.7	5.31 ± 0.38	‐
	6 m	8.96 ± 3.90	47.3 ± 10.3	5.03 ± 0.35	‐
Lower leg					
	Birth	5.91 ± 2.72	19.7 ± 4.6	6.70 ± 0.67	‐
	42 d	7.94 ± 4.04	33.4 ± 8.7	5.25 ± 0.39	‐
	6 m	8.58 ± 4.33	43.9 ± 10.2	4.78 ± 0.29	‐

*Note:* Data shown are mean ± standard deviation or median (quartile), as appropriate.

Abbreviations: TEWL, transepidermal water loss; SCH, stratum corneum hydration.

We found some differences in dynamic trends of the four parameter values between exposed and less‐exposed body sites. The postnatal rise in TEWL on exposed body sites (cheek and forehead) was greater than that on less‐exposed sites (abdomen, volar forearm, and lower leg) from birth to 42 days and declined in a faster rate to levels similar to those at birth by age 6 months (Table 2).

The SCH values showed an overall increasing trend from birth throughout early infancy, with a similar trend for exposed and less‐exposed body sites. Skin pH on all tested body sites and sebum content of the forehead showed an overall declining trend after birth until age 6 months, indicating overall acidification during early infancy (Table 2).

Sex differences were only found for sebum content on the forehead at birth or at 42 days, values in boys significantly higher than those in girls (*ps* < 0.001), but the difference became non‐significant at age 6 months (*p* = 0.57) (Table S1).

### 3.3 Trends of skin barrier parameters on the cheek and forearm among infants with and without AD

**FIGURE 2:**
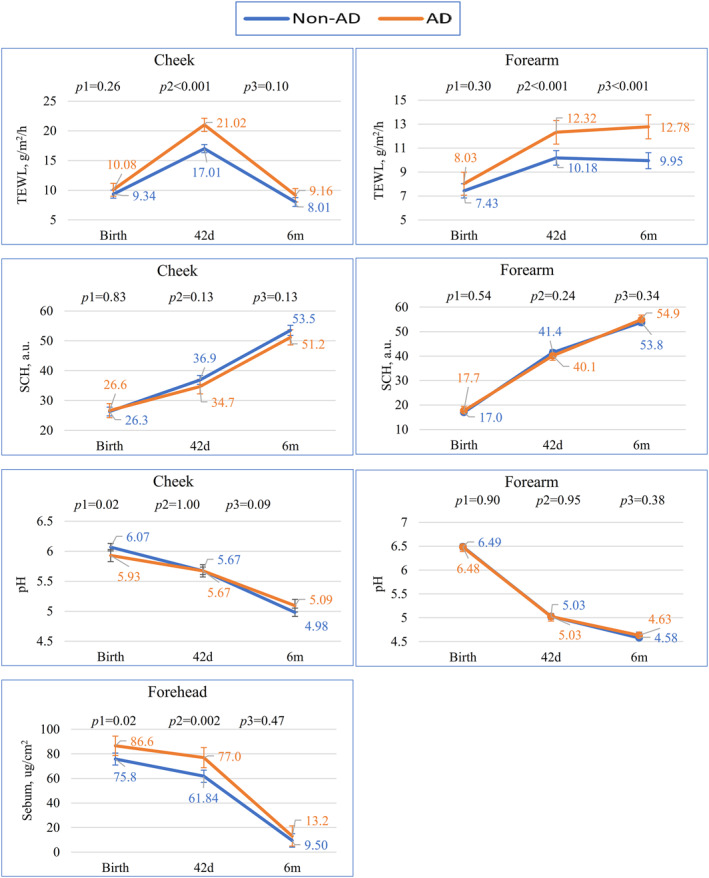
Differences in trends of four skin barrier parameters on the cheek and forearm in infants with and without Atopic dermatitis (AD). *p*1, *p*2 and *p*3 indicated significant between‐group differences at birth, 42 days, and 6 months, respectively, based on mixed models for repeated‐measures. Transepidermal water loss did not differ at birth but reached significantly higher levels in the AD group than in the non‐AD group at 42 days (21.02 vs. 17.01 g/m^2^/h on the cheek; 12.32 vs. 10.18 g/m^2^/h on the forearm, *p*2 < 0.001); only the significant difference on the forearm remained at 6 months (12.78 vs. 9.95, *p*3 < 0.001). Infants with AD had significantly higher sebum content at birth and 42 days, but the difference became smaller and did not significantly differ at 6 months. The trends pH or stratum corneum hydration over the first 6 months after birth did not differ between infants with and without AD

Mixed models for repeated measures showed significant differences in the dynamic trends of TEWL (*p* < 0.001), cheek pH (*p* = 0.03) and sebum content (*p* = 0.003) between the two groups, but not for SCH and forearm pH (Table S2). TEWL did not differ at birth, but TEWL reached higher levels in the AD group than the non‐AD group at 42 days (21.02 vs. 17.01 on the cheek; 12.32 vs. 10.18 on the forearm; *p* < 0.001). Only significant differences for the forearm TEWL remained at 6 months (12.78 vs. 9.95, *p* < 0.001). Cheek pH showed difference at birth (AD group 6.07 vs. non‐AD group 5.93), but not at 42 days and 6 months. Infants with AD had significantly higher sebum content values at birth and 42 days, but the difference became smaller and did not significantly differ at 6 months (Figure 2).

### 3.4 Associations of cheek and forearm skin barrier parameters among infants with AD

In univariate analysis, we found a weak association of AD risk within 1 year with cheek pH at birth, cheek TEWL values at 42 days, and forehead sebum content at 42 days. Additionally, the association of forearm TEWL at 42 days values with AD was significant (Table S3).

After adjusting for covariates such as parental allergy history, infant sex, birth weight, and feeding history, significant associations remained for cheek TEWL (OR = 1.26, 95% CI 1.00–1.57, *p* = 0.045 at birth, and OR = 1.52, 95% CI 1.17–1.97, *p* = 0.002 at 42 days). The results indicated that infants with higher cheek TEWL values by 1 SD at birth (4.0 g/m^2^/h) or at 42 days (7.8 g/m^2^/h) have increased risk of AD, by 26% and 52%, respectively; a similar association for forearm TEWL at 42 days with AD was also found (OR = 1.31, 95% CI 1.02–1.68, *p* = 0.032) (Table 3). No associations were not found between SCH, skin pH, and sebum content at birth or 42 days with AD risk age 1 year.

**TABLE 3:** Association of face or forearm skin parameter Z values for infant AD incidence within 1 year: multivariate models

Visit	Face (exposed)		Forearm (less exposed)	
	OR, 95%CI	*p* values	OR, 95%CI	*p* values
At birth				
TEWL	1.26, (1.00‐1.57)	**0.045**	1.10, (0.88‐1.37)	0.40
SCH	0.94, (0.71‐1.23)	0.64	0.81, (0.59‐1.10)	0.18
pH	0.81, (0.63‐1.05)	0.11	1.10, (0.84‐1.45)	0.49
Sebum content	1.00, (0.95‐1.04)	0.85		
At 42 days				
TEWL	1.52, (1.17‐1.97)	**0.002**	1.31, (1.02‐1.68)	**0.032**
SCH	1.03, (0.80‐1.33)	0.80	0.95, (0.73‐1.24)	0.71
pH	0.83, (0.63‐1.11)	0.21	1.06, (0.83‐1.37)	0.63
Sebum content	1.09, (0.97‐1.23)	0.13		

*Note:* Discrete time‐to‐event models were conducted by time and body part; four parameters were tested in the same model. Adjusted for infant’s sex, birth weight, birth season, delivery mode, feeding history, solid food introduction, maternal allergy history, and paternal allergy history. All the p values below 0.05 are bolded, indicating that the corresponding associations (OR and 95%CI) are statistically significant.

Abbreviations: AD, atopic dermatitis; CI, confidence interval; SCH, stratum corneum hydration; TEWL, transepidermal water loss; OR, odds ratio.

## 4 DISCUSSION

To our best knowledge, this study is the first to describe dynamic trends of four skin barrier function parameters (TEWL, SCH, skin pH, and sebum content) from birth to 6 months, based on a prospective cohort of full‐term Chinese infants. We found that infants' skin barrier functions showed different trends with age in response to exposure to the external environment. These postnatal changes in skin barrier functions within the first 6 months were generally non‐linear and diverse. TEWL increased from birth to 42 days, followed by a declining trend to birth level at 6 months. However, pH and sebum content showed an overall declining trend over the same period. SCH showed an increase after birth. Trends in the four parameters shared a common feature in that the most significant changes appeared during the first 4–6 weeks after birth, indicating a functional adaptation process in early life among these infants. Sex differences were only found for sebum content. We also found that TEWL values tested in the early stages of life were independently associated with AD risk, adding further evidence to our results.

As an indicator of water loss from the epidermis, TEWL is the most studied noninvasive skin barrier parameter.^31^ Neonatal TEWL values in our infants without AD were below 10 g/m^2^/h, which was in accordance with previous findings.^21^, ^32^ The dramatic increase in TEWL values on exposed body sites during the first 4–6 weeks of life might be partly explained by increasing superficial chymotrypsin‐like protease activity, which accords with rapid growth in infants.^19^ The greater increase of TEWL in exposed regions than less‐exposed regions might be associated with underlying differences in the physiological structure of the skin and speed of maturity during early life.^13^ As reported by McAleer et al. cheek natural moisturizing factor levels increase slower than in the elbow flexure, and cheek corneocyte envelope immaturity does not improve rapidly with age.^13^ Regional variation in the change of TEWL with growth supports the need to consider adjustment of our current infant skin care practices for different anatomical sites.

We found similar findings for the change trends of pH values, which showed a sharp decline in the postnatal 2 months and remained steady between 4.38 and 5.03 at 6 months, consistent with other studies^11^, ^19^ and the differences among anatomical sites at birth.^2^ The characteristic neutral skin surface pH at birth (5.74–6.70) might be owing to exposure to slightly alkaline amniotic fluid^33^; the sharp decline after birth might be partly explained by acid mantle formation during the neonatal period and related endogenous pathway comprising the sodium/hydrogen antiporter‐1 and secretory phospholipase A2, observed in a rat model.^34^

Previous studies reported that SCH increased significantly in the first 4 weeks after birth, and then remained stable^11^, ^18^, ^20^; however, we observed a constant increase of SCH from 42 days up to 6 months, implying that skin maturation persisted longer in this cohort. The differences in study design, ethnicity, and season of measurement might also be involved.

Little is known about the activity of sebaceous glands in neonates.^5^, ^23^ In our study, the sebum content declined dynamically with age, which might be owing to the role of transplacental hormone. At age 6 months, sebum content values are nearly too low to be detected. We report a significant sex difference, with higher levels in male infants than females over the first 6 months of life, which may be caused by differences in sexual hormone levels in early infancy.

TEWL was identified as the only predictor of subsequent AD risk among the four skin barrier parameters in our study. Every 1‐SD increment of cheek TEWL at birth (approximately 4.0 g/m^2^/h) and at 42 days (approximately 7.8 g/m^2^/h) was significantly associated with a 26% and 52% higher AD risk, respectively. In the study by Kikuchi et al. such association was only detected for TEWL at 3 months on forearm 21, small sample size (*n* = 24) limited the statistical power of the finding. Our finding was consistent with two earlier larger studies in European and Japanese populations.^28^, ^29^ In a larger prospective study by Kelleher et al. TEWL over the 75th percentile at age 2 months but not at birth was a significant predictor of infant AD at 1 year, independent of loss‐of‐function mutation in the *FLG* gene.^28^ These findings support the potential benefits of identifying infants with a high risk of developing AD in early life who can benefit from early intervention. Possible clinical strategies to maintain healthy skin TEWL levels in infants during the neonatal period might be helpful to reduce the risk of AD. Our findings provided stronger evidence in causal inference between skin water loss in early life and AD risk, although the underlying biological mechanism is not yet completely understood.

Our study has several strengths. To date, this is the first cohort study describing the dynamic trends of multiple skin barrier parameters (TEWL, SCH, skin pH, and sebum content) at five body sites during early life among healthy term infants and the first report of a linear association of TEWL with AD in Chinese infants.

This study also has some limitations. Preterm infants were not included, so interpretation and generalization of the study findings for preterm infants should be made with caution. Skin barrier function was examined in only three visits; variation in trends during a longer period, from 42 days to 6 months, might be biased. Additionally, 17.5% of infants in this cohort missed the 6‐month skin barrier test, which might introduce selection bias. The effect of established mutation of the *FLG* gene on infant AD risk was not investigated in our study.

## 5 CONCLUSION

In summary, we presented dynamic trends of four skin barrier parameters from birth to 6 months of age, and provided evidence from the Chinese infants that higher TEWL values one month after birth was predictive to increased risk of developing early‐onset AD. Our study added evidence on this topic and support that control of skin water loss during the first 2 months of life might benefit infants with potential higher risk of AD.

## AUTHOR CONTRIBUTION

Project management and study design: Ying Ye, Liuhui Wang and Weili Yan (principal investigator). Obstetrical data collection: Lei Zhang. Child care data collection: Yun Li. Skin barrier examination, skin disorder collection and AD diagnosis: Ying Ye, Limin Dou, Piaoping Zhao, Yalan Dou and Yuang Jiang. Data input and statistical analysis: Yi Zhang, Yalan Dou and Yuang Jiang. Manuscript drafting: Ying Ye, Liuhui Wang and Weili Yan conceived the study, supervised the statistical analyses and made the final approval of the manuscript. All authors contributed to data interpretation and reviewing the manuscript.

## Supporting information

Supporting Information S1
