# Supplementary material for: Dynamic trends in skin barrier function from birth to age 6 months and infantile atopic dermatitis: A Chinese prospective cohort study
Source: Clin Transl Allergy. 2021 Jul 3;11(5):e12043. doi: 10.1002/clt2.12043 (PMC8254580; doi:10.1002/clt2.12043)
Supplement: Supplementary file 1 — Supporting Information S1 [file CLT2-11-e12043-s001.docx]

Table S1. Sex differences in sebum content on forehead at birth, 42 days, and 6 months

| **Visit** | **Male** | | |  | **Female** | | |  | ***p* value** |
| --- | --- | --- | --- | --- | --- | --- | --- | --- | --- |
|  | **Mean** |  | **SE** |  | **Mean** |  | **SE** |  |  |
| Birth | 88.42 | ± | 3.39 |  | 64.54 | ± | 3.20 |  | <0.001 |
| 42d | 72.50 | ± | 3.45 |  | 52.55 | ± | 3.23 |  | <0.001 |
| 6 m | 8.13 | ± | 3.70 |  | 11.09 | ± | 3.66 |  | 0.57 |

Values are estimated mean ± standard error.

Differences by age were tested using mixed models among infants without atopic dermatitis only.

Table S2. Differences in skin barrier overall trend on the cheek and forearm in infants with or without Atopic dermatitis (AD)

|  | **Skin barrier parameter** | | | | | | | |
| --- | --- | --- | --- | --- | --- | --- | --- | --- |
| **Variable** | **Cheek, Coef (95%CI)** | | | |  | **Forearm, Coef (95%CI)** | | |
|  | **TEWL** | **SCH** | **pH** | **Sebum**† |  | **TEWL** | **SCH** | **pH** |
| **Group** | 0.74  (-0.53~2.01) | 0.3  (-2.5~3.1) | -0.14  (-0.26~-0.02)* | 10.8  (1.5~20.1)* |  | 0.60  (-0.53~1.73) | 0.7  (-1.4~2.7) | -0.007  (-0.12~0.11) |
| **Time** |  |  |  |  |  |  |  |  |
| 42 days | 7.67  (6.73~8.61)* | 10.6  (8.5~12.6)* | -0.40  (-0.48~-0.31)* | -13.9  (-20.6~-7.3)* |  | 2.75  (1.92~3.58)* | 24.4  (22.9~26.0)* | -1.46  (-1.54~-1.38)* |
| 6 months | -1.33  (-2.32~-0.34)* | 27.2  (25.0~29.4)* | -1.09  (-1.18~-0.99)* | -66.3  (-73.3~-59.2)* |  | 2.52  (1.64~3.39)* | 36.8  (35.1~38.4)* | -1.91  (-2.00~-1.83)* |
| **Interaction (group and time)** |  |  |  |  |  |  |  |  |
| Group#42 days | 3.27  (1.47~5.07)* | -2.5  (-6.5~1.4) | 0.14  (-0.03~0.31) | 4.3  (-8.4~17.1) |  | 1.54  (-0.03~3.11) | -2.0  (-4.9~1.0) | 0.004  (-1.15~0.16) |
| Group#6 months | 0.41  (-1.42~2.25) | -2.6  (-6.7~1.4) | 0.25  (0.08~0.42)* | -7.1  (-20.1~5.9) |  | 2.23  (0.63~3.84)* | 0.4  (-2.6~3.4) | 0.06  (-0.10~0.22) |
| **Constant** | 9.34  (8.67~10.01) | 26.3  (24.9~27.8) | 6.07  (6.00~6.13) | 75.8  (70.9~80.7) |  | 7.43  (6.84~8.03) | 17.0  (15.9~18.1) | 6.49  (6.43~6.55) |
| ***p* value for group difference (AD vs. non-AD)** | <0.001 | 0.20 | 0.03 | 0.003 |  | <0.001 | 0.42 | 0.85 |

Abbreviations: TEWL, transepidermal water loss; SCH, stratum corneum hydration; Coef, coefficient; CI, confidence interval.

†Sebum content was tested on forehead.

**p* value<0.05.

Mixed models for repeated measures were used to test the overall trend differences of skin barrier parameters (TEWL, pH, SCH and sebum content) between the AD and non-AD groups.

Table S3. Association of face or forearm skin parameter Z values with infant AD incidence within 1 year: univariate models

| **Visit** | **Face (exposed)** | |  | **Forearm (less exposed)** | |
| --- | --- | --- | --- | --- | --- |
|  | **OR, 95% CI** | ***p* value** |  | **OR, 95% CI** | ***p* value** |
| **At Birth** |  |  |  |  |  |
| TEWL | 1.17, 0.97-1.41 | 0.10 |  | 1.10, 0.92-1.32 | 0.28 |
| SCH | 0.91, 0.74-1.13 | 0.40 |  | 1.10, 0.89-1.36 | 0.38 |
| pH | 0.80, 0.65-0.99 | **0.039** |  | 1.02, 0.83-1.26 | 0.84 |
| Sebum content | 1.03, 0.99-1.07 | 0.07 |  | - | - |
| **At 42 days** |  |  |  |  |  |
| TEWL | 1.57, 1.24-1.98 | **<0.001** |  | 1.38, 1.10-1.73 | **0.006** |
| SCH | 0.93, 0.73-1.21 | 0.62 |  | 0.96, 0.75-1.22 | 0.73 |
| pH | 0.82, 0.63-1.08 | 0.15 |  | 1.02, 0.81-1.30 | 0.85 |
| Sebum content | 1.15, 1.05-1.28 | **0.004** |  | - | - |

Abbreviations: TEWL, transepidermal water loss; SCH, stratum corneum hydration; OR, odds ratio; CI, confidence interval.

Discrete time-to-event survival analyses were conducted by body part and by follow-up time respectively; four parameters were tested in the same model.
